# The Prevalence and Correlates of Probable Major Depressive Disorder and Probable Generalized Anxiety Disorder during the COVID-19 Pandemic. Results of a Nationally Representative Survey in Germany

**DOI:** 10.3390/ijerph182312302

**Published:** 2021-11-23

**Authors:** André Hajek, Hans-Helmut König

**Affiliations:** Hamburg Center for Health Economics, Department of Health Economics and Health Services Research, University Medical Center Hamburg-Eppendorf, 20246 Hamburg, Germany; h.koenig@uke.de

**Keywords:** prevalence, depression, anxiety, Patient Health Questionnaire (PHQ), generalized anxiety disorder, mental disorders, SARS-CoV-2, COVID-19

## Abstract

The aim was to clarify the prevalence and correlates of probable major depressive disorder and probable generalized anxiety disorder in the general adult population during the COVID-19 pandemic. Data were derived from a nationally representative survey (August and September 2021). In total, *n* = 3075 individuals took part. To quantify probable generalized anxiety disorder, the established Generalized Anxiety Disorder-7 (GAD-7; cutoff of 10) was used. Moreover, the Patient Health Questionnaire-9 (PHQ-9; cutoff of 10) was used to quantify probable major depressive disorder. The prevalence of probable major depressive disorder was 20.0% and the prevalence of probable generalized anxiety disorder was 13.4%. Particularly high prevalence rates were observed for younger individuals, individuals with migration background and individuals with at least one chronic disease. The likelihood of probable major depressive disorder was positively associated with younger age, being unmarried, having a migration background, smoking, daily alcohol intake, the presence of chronic diseases and lower self-rated health. Similarly, the likelihood of probable generalized anxiety disorder was positively associated with younger age, being unmarried, smoking, the presence of chronic diseases and lower self-rated health. In conclusion, the magnitude of probable major depressive disorder and probable generalized anxiety disorder in Germany in late summer of 2021 was highlighted. Identifying the correlates of them may help to tackle individuals at higher risk.

## 1. Introduction

High prevalence rates of depression and anxiety have repeatedly been shown in the past [1,2]. In times of the current pandemic, individuals are confronted with several challenges including social distancing [3] and economic uncertainties. According to a meta-analysis (including general populations globally during the pandemic), the prevalence of anxiety and depression was 32% (95% CI: 28–37) and 34% (95% CI: 28–41), respectively [4]. Globally, it should be emphasized that most of the studies used convenience samples and only some studies using nationally representative samples (from a specific country, e.g., United Kingdom, Austria, United States, Australia, Scotland or Republic of Ireland) during the pandemic (e.g., [5,6,7,8,9,10,11,12,13,14,15]).

Furthermore, globally, most of the existing studies focused on the pandemic in the year 2020, whereas only a few nationally representative studies exist identifying the prevalence and correlates of probable major depressive disorder and probable generalized anxiety disorder in the year 2021.

This is of importance because vaccinations against COVID-19 have been available since the end of 2020 (in various countries including Germany). These vaccinations are recommended by, for example, the Food and Drug Administration, the European Medicines Agency and by the Standing Committee on Vaccination at the Robert Koch Institute (Germany).

Consequently, our objective was to identify the prevalence and correlates of probable major depressive disorder and probable generalized anxiety disorder in the general adult population during the COVID-19 pandemic (late summer of 2021). Knowledge regarding the correlates of these disorders may assist in characterizing at-risk individuals. This in turn may be beneficial to reduce the likely rise in these mental disorders [16].

## 2. Materials and Methods

### 2.1. Sample

For our current study, data were used from a nationally representative online survey covering individuals aged 18 to 70 years and living in Germany (*n* = 3075). Thus, the only inclusion criteria were the following: aged 18 to 70 years and residing in Germany. This also means that individuals aged 17 and younger and individuals aged 71 years and over were excluded. Moreover, individuals not residing in Germany were excluded. Other exclusion criteria did not exist. However, the questionnaire was only available in the German language. Thus, it is most likely that individuals who could not read German did not participate.

The survey took place from 24 August to 3 September 2021. The participants were recruited by a well-known market research company, which owns an online access panel. For this online sample, multiple recruitment sources were used (e.g., opinion platforms, search engine marketing or cooperation agreements).

Respondents were drawn from this online sample in a way that corresponded to the distribution of age group, gender and federal state in the general German population [17] (based on these sociodemographic data, a random sample from the population of the online access panel was drawn). The quotas were from Best for Planning 2020. About 14,000 individuals were invited to participate. There was a maximum of two reminders after the initial invitation mail (thus, three mails in total). The interval between two reminders was at least two days. To avoid duplicates (i.e., multiple registrations from the same individual), digital fingerprint solutions were used.

Participants provided informed consent. The study was approved by the Local Psychological Ethics Committee of the Center for Psychosocial Medicine of the University Medical Center Hamburg-Eppendorf (number: LPEK-0356).

### 2.2. Outcome

Patient Health Questionnaire-9 (PHQ-9) was used to quantify probable major depressive disorder. It is a widely used (e.g., [18]) and valid tool consisting of nine items [19]. As an example, individuals were asked, how often they feel bothered by “little interest or pleasure in doing things” in the past two weeks (not at all; several days; more than half of the days; nearly every day). A sum score was computed ranging from 0 to 27, with higher scores indicating more depressive symptoms. Based on a PHQ-9 score of ≥10, the sensitivity was 0.88 and the specificity was 0.88 for major depressive disorder [19]. In our study, Cronbach’s alpha was 0.91. In line with previous studies, a cutoff of ≥10 was used in our study [20].

Generalized Anxiety Disorder-7 (GAD-7; cutoff of 10) was used to assess probable generalized anxiety disorder [21]. It consists of seven items. A sum score was calculated (from 0 to 21), with higher scores indicating more anxiety symptoms. The sensitivity was 0.89 and the specificity was 0.82 (when GAD-7 score ≥ 10). In our study, Cronbach’s alpha was 0.92. Based on the existing recommendations [21], the cutoff of ≥10 was used in our study.

### 2.3. Independent Variables

As correlates, we included the following in regression analysis (all were self-reported): gender, age, marital status (married, living together with spouse; married, not living together with spouse; widowed; divorced; single), presence of children in the same household (no or yes), migration background (no or yes), highest educational degree (upper secondary school; qualification for applied upper secondary school; polytechnic secondary school; intermediate secondary school; lower secondary school; currently in school training/education; without school-leaving qualification) and employment status (full-time employed; retired; other).

Additionally, we included smoking behavior (yes, daily; yes, sometimes; no, not anymore; never smoker), alcohol consumption (daily; several times per week; once a week; 1–3 times per month; less often; never), and sports activities (no sports activity; less than one hour a week; regularly, 1–2 h a week; regularly, 2–4 h a week; regularly, more than 4 h a week). Additionally, vaccinated against COVID-19 (no or yes), chronic diseases (absence of chronic diseases; presence of one or more chronic diseases; exact wording: “Do you have at least one chronic condition?”; no or yes) and self-rated health (exact wording: “How would you rate your current health?”; 1 = very bad; 2 = poor; 3 = average; 4 = good; 5 = very good) were included as covariates.

### 2.4. Statistical Analysis

First, prevalence rates are displayed (also stratified by several sociodemographic factors such as age group and health-related factors). Thereafter, multiple logistic regressions were conducted (first regression: with probable major depressive disorder as the outcome measure; second regression: with probable generalized anxiety disorder as the outcome measure). It should be noted that we dichotomized the marital status (0: married, not living together with spouse; widowed; divorced; single; 1: married, living together with spouse). The level of significance was set at *p* < 0.05. Stata 16.1 (Stata Corp., College Station, TX, USA) was used for performing statistical analyses.

## 3. Results

### 3.1. Key Sample Characteristics and Prevalence Rates

In our sample, the average age equaled 44.5 years (SD: 14.8 years). Furthermore, 51.1% of the participants were female. A comparison of the target cohort and our sample is given in Supplementary Table S1. Prevalence rates for probable major depressive disorder and probable generalized anxiety disorder (also stratified by several sociodemographic and health-related factors) are displayed in Table 1.

The prevalence of probable major depressive disorder was 20.0% and the prevalence of probable generalized anxiety disorder was 13.4%. Particularly high prevalence rates were identified among younger individuals aged 18 to 29 years (probable major depressive disorder: 31.8%; probable generalized anxiety disorder: 23.1%), among individuals with migration background (probable major depressive disorder: 30.2%; probable generalized anxiety disorder: 19.4%) and among individuals with at least one chronic disease (probable major depressive disorder: 27.5%; probable generalized anxiety disorder: 17.5%).

### 3.2. Regression Analysis

The findings of multiple logistic regressions are shown in Table 2.

The likelihood of probable major depressive disorder was positively associated with younger age (OR: 0.94, 95% CI: 0.93–0.95), being unmarried (OR: 0.73, 95% CI: 0.58–0.90), having a migration background (OR: 1.60, 95% CI: 1.19–1.60), smoking (e.g., “Yes, sometimes” compared to “never smoker”, OR: 1.85, 95% CI: 1.28–2.66), daily alcohol intake (compared to “never”, OR: 1.63, 95% CI: 1.01–2.64), the presence of chronic diseases (OR: 1.78, 95% CI: 1.40–2.25) and lower self-rated health (OR: 0.29, 95% CI: 0.25–0.34).

The likelihood of probable generalized anxiety disorder was positively associated with younger age (OR: 0.94, 95% CI: 0.93–0.95), being unmarried (OR: 0.77, 95% CI: 0.60–0.99), smoking (“Yes, sometimes” compared to “never smoker”, OR: 2.16, 95% CI: 1.45–3.22), the presence of chronic diseases (OR: 1.44, 95% CI: 1.09–1.88) and lower self-rated health (OR: 0.29, 95% CI: 0.25–0.34).

## 4. Discussion

### 4.1. Main Findings

Using data from the general adult population, our goal was to clarify the prevalence and correlates of probable major depressive disorder and probable generalized anxiety disorder in late summer of 2021. While the prevalence of probable major depressive disorder was 20.0%, the prevalence of probable generalized anxiety disorder was 13.4%. Particularly high prevalence rates were identified among younger individuals aged 18 to 29 years, among individuals with migration background and among individuals with at least one chronic disease. Some groups (gender: diverse; without school-leaving qualification) also reported very high prevalence rates. However, sample sizes were very small for these groups and these findings should, therefore, be treated with great caution.

Similar correlates were identified for both probable major depressive disorder and probable generalized anxiety disorder (e.g., younger age, lifestyle factors and health-related factors).

### 4.2. Previous Research and Possible Explanations

Our study showed that probable major depressive disorder and probable generalized anxiety disorder are frequent in the general adult population in Germany. These prevalence rates were higher compared to almost ten years ago (based on the PHQ-4 cutoff values: 10.4% of the individuals had probable depression and 9.8% of the individuals had probable anxiety) [22]. However, compared to April 2021, prevalence rates were slightly lower for probable major depressive disorder (23.7%), whereas they were markedly lower for probable generalized anxiety disorder in Germany (22.3%) [23]. This may be explained by differences in the tools used (GAD-2 versus GAD-7). Moreover, individuals may have adapted to the conditions of the pandemic and may have overcome times of economic challenges, health concerns and loneliness.

The highest prevalence rates were identified among young adults, individuals with migration background and individuals with one or more chronic diseases. In particular, young adults may be confronted with challenges such as financial hardships or concerns about the future. Similar findings were made by Ranta et al. in Finland [24]. Such factors may explain their high prevalence rates. Individuals with chronic diseases may fear a potential severe course of an infection with the corona virus. Therefore, they may have comparably high prevalence rates. Moreover, individuals with a migration background may have high prevalence rates since they may be concerned about friends and relatives who may live abroad. They may fear that they cannot visit these friends and relatives in case of emergency due to potential travel restrictions. Such concerns were also described by a recent qualitative study examining different migrant groups living in Finland during the pandemic [25]. Additionally, fear due to insufficient knowledge about the coronavirus (resulting from language barriers) may also explain the comparably high prevalence rates. Apart from that, the correlates of probable major depressive disorder and probable generalized anxiety disorder (e.g., sociodemographic factors, lifestyle-related factors and health-related factors) during late summer 2021 are well in line with previous studies (e.g., [26]). For example, previous research also demonstrated associations between cigarette smoking [27] or marital status [28] and our outcomes.

### 4.3. Strengths and Limitations

Some strengths and limitations are worth acknowledging. Data came from a representative sample (collected in late summer of 2021). Both outcomes are valid, and there are well-established tools to quantify probable major depressive disorder and probable generalized anxiety disorder. However, upcoming studies based on clinical interviews are preferable to confirm our findings. Moreover, future longitudinal studies are required. It should be noted that our online survey was only available in the German language. Moreover, potential differences between non-respondents and respondents could not be calculated.

## 5. Conclusions and Future Research

Our current study extends our knowledge regarding the level of probable major depressive disorder and probable generalized anxiety disorder in the general adult population in Germany. The magnitude of probable major depressive disorder and probable generalized anxiety disorder in Germany in late summer 2021 was highlighted. Identifying the correlates of these disorders may help to tackle individuals at higher risk. Ultimately, this knowledge may assist in reducing the projected rise in mental disorders [16]. Future research is required in this area, for example, based on longitudinal data. Moreover, future research is required to clarify what consequences this expected increase in mental disorders has for different outcomes (e.g., morbidity and mortality).

## Figures and Tables

**Table 1:** Prevalence rate for major depressive disorder and probable generalized anxiety disorder among several groups.

	*n*	Presence of Probable Major Depressive Disorder	Presence of Probable Generalized Anxiety Disorder
Total sample	3075	614 (20.0%)	412 (13.4%)
Gender			
Male	1502	249 (16.6%)	164 (10.9%)
Female	1570	363 (23.1%)	247 (15.7%)
Diverse	3	2 (66.7%)	1 (33.3%)
Age group			
18 to 29 years	628	200 (31.8%)	145 (23.1%)
30 to 39 years	597	126 (21.1%)	87 (14.6%)
40 to 49 years	597	105 (17.6%)	72 (12.1%)
50 to 59 years	659	113 (17.1%)	72 (10.9%)
60 years and older	594	70 (11.8%)	36 (6.1%)
Children in own household			
No	2206	461 (20.9%)	311 (14.1%)
Yes	869	153 (17.6%)	101 (11.6%)
Marital status			
Single/divorced/widowed/married, not living together with spouse	1313	340 (25.9%)	230 (17.5%)
Married, living together with spouse	1762	274 (15.6%)	182 (10.3%)
Education			
Upper secondary school	1326	251 (18.9%)	183 (13.8%)
Qualification for applied upper secondary school	328	66 (20.1%)	38 (11.6%)
Polytechnic secondary school	168	23 (13.7%)	12 (7.1%)
Intermediate secondary school	888	192 (21.6%)	119 (13.4%)
Lower secondary school	347	75 (21.6%)	55 (15.9%)
Currently in school training/education	9	2 (22.2%)	1 (11.1%)
Without school-leaving qualification	9	5 (55.6%)	4 (44.4%)
Migration background			
No	2724	508 (18.6%)	344 (12.6%)
Yes	351	106 (30.2%)	68 (19.4%)
Employment status			
Full-time employed	1458	237 (16.3%)	166 (11.4%)
Retired	499	86 (17.2%)	48 (9.6%)
Other	1118	291 (26.0%)	198 (17.7%)
Vaccinated against COVID-19			
No	593	130 (21.9%)	98 (16.5%)
Yes	2482	484 (19.5%)	314 (12.7%)
Chronic diseases			
Absence of at least one chronic disease	1765	254 (14.4%)	183 (10.4%)
Presence of at least one chronic disease	1310	360 (27.5%)	229 (17.5%)

**Table 2:** Correlates of probable major depressive disorder and probable generalized anxiety disorder. Results of multiple logistic regressions.

Independent Variables		Probable Major Depressive Disorder	Probable Generalized Anxiety Disorder
Sex:	- Women (Ref.: men)	1.18	1.24
		(0.94–1.49)	(0.95–1.61)
	- Diverse	2.76	0.71
		(0.19–41.01)	(0.04–14.50)
Age (in years)		0.94 ***	0.94 ***
		(0.93–0.95)	(0.93–0.95)
Children in own household:	- Yes (Reference: no)	0.91	0.89
		(0.71–1.16)	(0.67–1.18)
Marital status:	- Married, living together with spouse (Ref.: single/divorced/widowed/married, not living together with spouse)	0.73 **	0.77 *
		(0.58–0.90)	(0.60–0.99)
Highest educational degree:	- Qualification for applied upper secondary school (Ref.: upper secondary school)	1.00	0.72
		(0.71–1.42)	(0.47–1.09)
	- Polytechnic secondary school	0.74	0.53 +
		(0.43–1.26)	(0.27–1.06)
	- Intermediate secondary school	1.09	0.88
		(0.84–1.41)	(0.65–1.18)
	- Lower secondary school	1.14	1.26
		(0.79–1.64)	(0.84–1.88)
	- Currently in school training/education	0.85	0.58
		(0.14–4.99)	(0.06–5.16)
	- Without school-leaving qualification	1.56	1.62
		(0.35–6.84)	(0.37–7.10)
Migration:	- Migration background (Ref.: no migration background)	1.60 **	1.26
		(1.19–2.15)	(0.90–1.75)
Employment status:	- Retired (Ref.: full-time employed)	0.96	0.76
		(0.67–1.38)	(0.49–1.17)
	- Other	1.05	0.96
		(0.83–1.33)	(0.74–1.25)
Smoking:	- Yes, daily (Ref: never smoker)	1.36 *	1.25
		(1.03–1.80)	(0.91–1.73)
	- Yes, sometimes	1.85 ***	2.16 ***
		(1.28–2.66)	(1.45–3.22)
	- No, not anymore	1.24	1.13
		(0.94–1.63)	(0.82–1.55)
Sports activities:	- Less than one hour a week (Ref.: no sports activity)	1.18	1.04
		(0.89–1.57)	(0.75–1.46)
	- Regularly, 1–2 h a week	1.20	1.18
		(0.89–1.60)	(0.84–1.65)
	- Regularly, 2–4 h a week	0.96	1.25
		(0.68–1.37)	(0.84–1.84)
	- Regularly, more than 4 h a week	0.90	1.20
		(0.61–1.33)	(0.78–1.84)
Alcohol intake:	- Daily (Ref.: never)	1.63 *	1.45
		(1.01–2.64)	(0.83–2.53)
	- Several times a week	1.30	1.33
		(0.92–1.85)	(0.90–1.97)
	- Once a week	1.18	1.25
		(0.83–1.69)	(0.84–1.87)
	- 1–3 times a month	0.95	0.82
		(0.68–1.33)	(0.55–1.21)
	- Less often	1.02	0.85
		(0.75–1.38)	(0.60–1.21)
Vaccinated against COVID-19:	- Yes (Ref.: no)	1.12	0.91
		(0.86–1.45)	(0.68–1.22)
Chronic diseases:	- Presence of at least one chronic disease (absence of chronic diseases)	1.78 ***	1.44 **
		(1.40–2.25)	(1.09–1.88)
Self-rated health (1 = very bad to 5 = very good)		0.29 ***	0.29 ***
		(0.25–0.34)	(0.25–0.34)
Constant		110.89 ***	89.08 ***
		(49.52–248.31)	(36.32–218.52)
Pseudo R²		0.22	0.20
Observations		3075	3075

Odds ratios are displayed; 95% confidence intervals in parentheses; *** *p* < 0.001, ** *p* < 0.01, * *p* < 0.05, + *p* < 0.10; continuous variables included in regression analysis were as follows: age and self-rated health. All other independent variables were nominal or categorical.

## Data Availability

The datasets used and analyzed during the current study are available from the corresponding author on reasonable request for all interested researchers.

## Supplementary Materials

The following are available online at https://www.mdpi.com/article/10.3390/ijerph182312302/s1, Table S1: Comparison of the target quote and our sample.
