# Supplementary material for: The Prevalence and Correlates of Probable Major Depressive Disorder and Probable Generalized Anxiety Disorder during the COVID-19 Pandemic. Results of a Nationally Representative Survey in Germany
Source: Int J Environ Res Public Health. 2021 Nov 23;18(23):12302. doi: 10.3390/ijerph182312302 (PMC8656556; doi:10.3390/ijerph182312302)
Supplement: Supplementary file 1 [file ijerph-18-12302-s001.zip › ijerph-1431273-supplementary.pdf]

Supplementary Material:

Table S1. Comparison of the target quote and our sample.

Sex	***Target quote***	***Our sample***
Men	50%	49%
Women	50%	51%
Age group		
18 – 29 years	20%	21%
30 – 39 years	19%	19%
40 – 49 years	19%	19%
50 – 59 years	21%	22%
60 – 70 years	21%	19%
State		
Baden-Wuerttemberg	13%	13%
Bavaria	16%	16%
Berlin	4%	4%
Brandenburg	3%	3%
Bremen	1%	1%
Hamburg	2%	2%
Hesse	7%	7%
Mecklenburg-Western Pomerania	2%	2%
Lower Saxony	10%	10%
North Rhine-Westphalia	22%	22%
Rhineland-Palatinate	5%	5%
Saarland	1%	1%
Saxony	5%	5%
Saxony-Anhalt	3%	5%
Schleswig-Holstein	3%	3%
Thuringia	3%	3%
